# Paneugenesis: a regenerative systems hypothesis for advancing health promotion

**DOI:** 10.3389/fpubh.2026.1823318

**Published:** 2026-05-29

**Authors:** Craig M. Becker, Leslie Hoglund, Beth Chaney, Joseph G. L. Lee, Michael Stellefson, Alex Davis

**Affiliations:** 1 Department of Health Education & Promotion, East Carolina University, Greenville, NC, United States; 2 Joint School of Public Health at Old Dominion University, Norfolk State University, Norfolk, VA, United States; 3 Department of Health Science, The University of Alabama, Tuscaloosa, AL, United States; 4 Department of Implementation Science, Wake Forest University School of Medicine, Winston-Salem, NC, United States; 5 Department of Music, East Carolina University, Greenville, NC, United States

**Keywords:** behavior change, health promotion theory, paneugenesis, quality improvement, regenerative systems, salutogenesis, systems science

## Abstract

Despite major advances in health promotion science, dominant approaches remain largely prevention-and risk-reduction-oriented. The prevailing orientation creates a substantial opportunity to advance generative, system-design strategies that intentionally produce well-being rather than merely prevent disease. This article proposes *paneugenesis* as a regenerative systems framework and a testable theoretical model for the intentional creation of net-positive outcomes, thereby extending health promotion beyond its traditional pathogenic emphasis. Paneugenesis integrates systems science, salutogenesis, behavioral science, complexity theory, and quality management principles into a unified four-function process: operationalizing idealized outcomes, identifying key precursors, optimizing processes, and continually plotting progress through feedback mechanisms. The central hypothesis is that health promotion systems explicitly designed to generate net-positive “+3 outcomes” (simultaneous benefits for [1] individuals, [2] others, and the [3] environment), as operationalized through paneugenesis principles, will demonstrate greater long-term improvements in validated well-being and health behavior measures compared to systems designed primarily around risk reduction. This framework draws on converging empirical evidence from behavioral and systems sciences and aligns with validated measurement tools, including the Salutogenic Wellness Promotion Scale (SWPS), which operationalizes regenerative engagement across multiple life domains. We describe the theoretical foundations, explain how paneugenesis is expected to work, present testable propositions, and discuss implications for research, measurement, policy, and system design. As a hypothesis and theory contribution, this article advances regeneration as a scientifically grounded and empirically testable evolution in health promotion science.

## 1 Introduction: the need for a regenerative theory of health promotion

A growing convergence across scientific fields shows that thriving can be intentionally created through well-designed, energy-adding, mutually reinforcing systems. Systems science shows that outcomes arise from structure and interaction patterns rather than from isolated individual choices (1, 2). Salutogenesis holds that health emerges from strengthening resources and coherence rather than merely reducing risk exposure (3–5). Behavioral science shows that beneficial actions become durable when environments and feedback systems make them easier and more meaningful to perform (6, 7). Complexity science shows that open systems develop higher levels of order when energy and information flows are properly organized (8). Together, these advances indicate that health and well-being can be intentionally designed rather than emerging solely as a by-product of problem-focused programs.

Contemporary health promotion scholarship suggests that the determinants of health and well-being in the 21st century increasingly require intentional system design that enables flourishing rather than solely preventing disease (9). Harari (10) similarly frames this shift as a broader societal transition toward designing systems that enable human flourishing. This reframing positions health promotion as a generative rather than merely protective discipline. This shift is consistent with Toffler's (11) observation that each era demands new tools, new assumptions, and new designs. The convergence of systems science, salutogenesis, behavioral science, and regenerative thinking now makes a further evolution of health promotion not only possible but necessary.

A further implication of converging systems and evolutionary science is that actions within interconnected systems are not neutral. Wright’s Nonzero framework shows that durable progress emerges when interactions generate mutual benefit rather than isolated gain, because reinforcing positive exchanges increases total system capacity over time (12). Actions that create benefits in one domain while externalizing costs to others tend to erode long-term stability. We refer to mutually reinforcing, net-positive interaction patterns as interbeneficence, the principle that system components improve their own outcomes by contributing to the improvement of other components (12, 13). From this perspective, health promotion actions fall along a continuum from net-negative to net-positive system effects, and designs that intentionally increase interbeneficent outcomes are more likely to produce sustained advancement. This systems orientation parallels prior descriptions of a “health-promoting ecosystem” in which interacting components generate reciprocal and multiplicative benefits across individuals, communities, and environments (14).

This integrative systems view is especially important because many of today’s major challenges appear distinct yet share common structural origins. Chronic illness, climate instability, social isolation, loss of meaning, and persistent inequity are often treated as separate problems, yet each reflects system conditions in which key relationships, feedback loops, and resource flows are misaligned or degraded. When behavioral, social, economic, and environmental systems are organized around short-term or siloed gains, they tend to generate downstream health, social, and ecological costs. Repeated population studies showing only limited improvement in health behaviors reinforce the need to revisit prevailing design approaches (15). In contrast, when systems are organized to produce reinforcing benefits across domains, improvements in one area strengthen others (1, 2, 12). This shared-condition pattern suggests that solutions should be system-integrated and mutually beneficial by design.

Prevention and risk reduction remain essential to health promotion and have produced important gains (16). However, systems designed primarily to avoid harm do not always generate the positive conditions that enable thriving. Additional design logic is needed to intentionally generate well-being, capacity, and resilience. This need points to the next step in health promotion: regeneration. Regeneration is the intentional creation of conditions that improve the present well-being while simultaneously increasing future capacity. It is neither the restoration of a prior state nor mere sustainability, but the design of systems that make life more livable over time (17–21).

This shift is consistent with prior work proposing a “chronic wellness” orientation, in which health is understood as a persistent, positive condition generated through engagement in health-promoting actions rather than the absence of disease (14). In this view, systems that cultivate health-promoting environments produce prevention as a by-product of strengthened capacity, rather than as the primary mechanism of change.^1^

**Figure 1:**
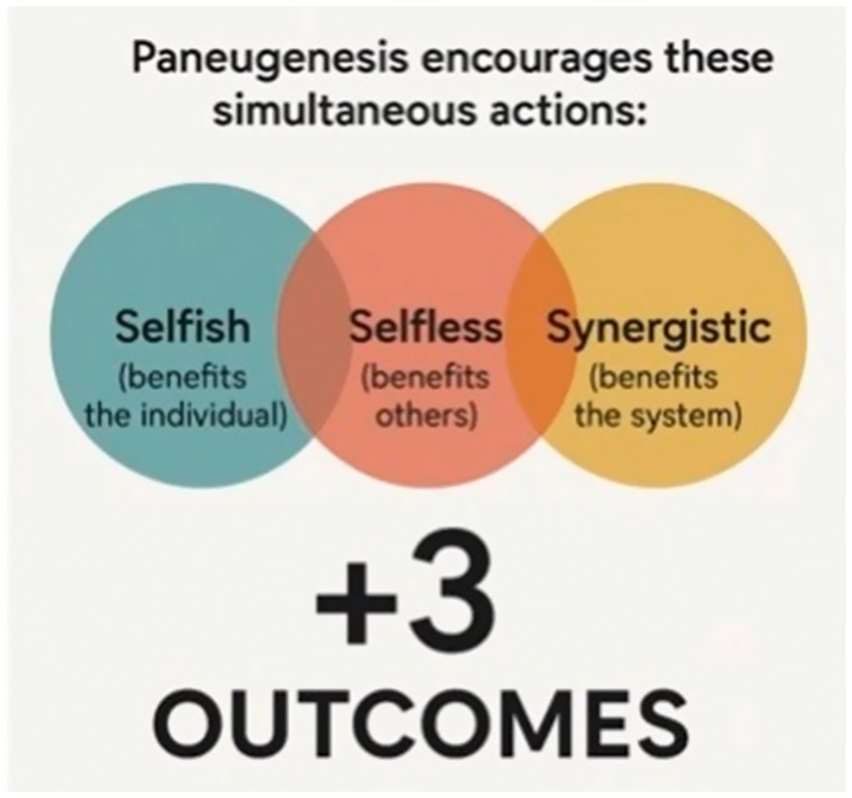
+3 outcomes in paneugenesis. Regenerative actions simultaneously benefit the self, others, and the environment, producing net-positive outcomes by design. These interdependent outcomes operationalize interbeneficence and define success as reinforcing, cross-domain improvement.

This article advances a formal framework for operationalizing regeneration in health promotion. We propose paneugenesis as a regenerative systems hypothesis and theory that specifies how systems can be intentionally designed to generate net-positive outcomes through structured processes, measurable precursors, and continual feedback (2, 22). The term paneugenesis derives from the Greek roots pan (all), eu (good), and genesis (creation), referring to the intentional creation of conditions that generate comprehensive benefits. In this framework, paneugenesis describes the intentional design of systems that generate pervasive, reciprocal, net-positive, regenerative interactions that are simultaneously selfish and selfless in their effects, creating synergistic benefits for individuals, others, and the environment. These mutually reinforcing outcomes are referred to as +3 outcomes, indicating simultaneous benefit across personal, social, and ecological domains (Figure 1). While paneugenesis is presented as a systems-level framework, it is designed to function across a range of implementation conditions, including contexts where system redesign authority is partial or constrained.

Paneugenesis frames health as an outcome that can be intentionally produced, rather than something that must merely be protected from loss. The theory centers on +3 outcomes, actions, and systems that simultaneously benefit individuals, others, and the environment (i.e., the +3), aligning with interbeneficence and nonzero system dynamics (12, 13). It integrates advances in systems science, salutogenesis, behavioral science, complexity theory, and quality management into a unified four-step model: operationalizing the idealized outcome, discovering precursors, optimizing processes, and plotting progress. The framework generates explicit, testable propositions and aligns with validated behavioral measures, including the Salutogenic Wellness Promotion Scale (SWPS), enabling empirical evaluation (23–25). Paneugenesis differs from existing models by specifying a unified, testable system design mechanism that generates health through reinforcing, cross-domain interactions rather than reducing isolated risks.

We advance the hypothesis that health, well-being, and ecological benefits can be achieved more reliably and sustainably when systems are intentionally designed to generate reinforcing, net-positive (+3) outcomes across personal, social, and environmental domains. That is, systems organized around mutually beneficial interactions and feedback-rich design will produce more durable improvements in engagement, well-being, and adaptive capacity than systems whose primary success criteria are risk reduction and problem control. This hypothesis is falsifiable and can be tested using comparative designs that evaluate behavioral precursors (e.g., SWPS), well-being outcomes, and system-level indicators over time.

The purpose of this Hypothesis & Theory article is to define the paneugenesis framework, articulate its scientific foundations, specify its mechanism model, and present testable propositions for future research. In doing so, it advances regeneration as a scientifically grounded and empirically evaluable next step in the evolution of health promotion, one that complements and extends, rather than replaces, the essential contributions of prevention and salutogenesis. This positions paneugenesis as both a theoretical extension and a practical framework for advancing health promotion. Paneugenesis proposes a testable systems design method for intentionally generating reinforcing improvements in health, social, and environmental outcomes.

## 2 Foundations of the paneugenesis hypothesis

The paneugenesis hypothesis builds on established foundations in salutogenesis and quality systems science. This perspective aligns with established implementation science frameworks that emphasize the importance of system context, structure, and feedback in determining outcomes (26, 27). Antonovsky’s salutogenic model shows that health emerges from strengthening resources and precursors of coherence rather than from a narrow focus on disease prevention (3, 5). Deming’s work holds that outcomes result from system design and continual process improvement rather than from isolated corrective action (2). Paneugenesis extends these foundations by specifying regenerative design criteria that aim for net-positive, better-than-before outcomes. While salutogenesis emphasizes the resources and conditions that generate health, and quality improvement provides methods for refining processes over time, paneugenesis integrates these traditions by specifying a unified system design mechanism through which idealized outcomes, behavioral precursors, process optimization, and measurement feedback operate together to intentionally generate reinforcing, cross-domain improvements.

Within this framework, regeneration is defined as the intentional creation of conditions that improve systems rather than merely restoring or sustaining them (20, 21). Preventive approaches reduce harm, whereas regenerative approaches produce measurable improvement.

Traditional and prevailing health promotion methods often emphasize positive aims but are frequently evaluated with prevention-centered metrics that define success primarily as reduced risk or avoided harm (16). Paneugenesis proposes a stronger criterion: success should be defined as gains in engagement, well-being, and functional capacity that are better than before. Empirical studies using the SWPS support this generative measurement approach, showing that higher engagement in health-promoting actions is associated with greater well-being and fewer reported problems (23–25).

Paneugenesis shares conceptual ground with several established frameworks but is distinguishable by its integration of generative success criteria, cross-domain outcome design, and an operational four-function mechanism with validated measurement. Salutogenesis identifies the origins of health but does not specify a design mechanism for intentionally generating new capacity. Quality improvement cycles provide process structure but do not define health-specific outcome criteria or require cross-domain benefit. Asset-based community development identifies existing strengths but does not specify how to design systems that generate new capacity or plot progress through feedback. Health promotion (Ottawa Charter) broadens scope through supportive environments and protective behaviors but is primarily evaluated using risk-reduction and disease-prevention metrics. The Social Ecological Model maps multi-level influences but does not specify how to design interactions across levels to generate reinforcing outcomes. Paneugenesis addresses each of these gaps by combining a generative success criterion (+3 outcomes), an interbeneficence-based design logic, and a repeatable four-function mechanism structured for empirical testing. To clarify how paneugenesis extends rather than duplicates existing health promotion and systems frameworks, Table 1 presents a direct conceptual comparison across primary questions, success criteria, design logic, outcome scope, and measurement approach.

**Table 1:** Comparison of paneugenesis with related frameworks.

Framework	Primary question	Success criterion	Design logic	Outcome scope	Measurement approach
Salutogenesis	What generates health?	Strengthened sense of coherence	Identify and build health resources	Individual resilience and adaptive capacity	Sense of coherence scales
Quality improvement (Deming)	How do we improve processes?	Reduced variation; continual improvement	Feedback-driven process refinement	Organizational or system performance	Process and outcome metrics
Asset-based community development	What strengths already exist?	Mobilized community assets	Identify and connect existing resources	Community capacity	Asset mapping
Health promotion (Ottawa Charter)	How do we build healthy environments and behaviors?	Improved health behaviors and reduced risk and increased protective behaviors	Multi-strategy enabling and mediating	Individual through policy levels	Risk reduction and behavioral indicators
Social ecological model	Where do health influences occur?	Reduced risk across ecological levels	Describe multi-level determinants	Individual through policy levels	Multi-level behavioral and environmental measures
Paneugenesis	How do we intentionally design systems to generate reinforcing better-than-before outcomes?	Measurable better-than-before gains across domains (+3 outcomes)	Four-function regenerative mechanism: operationalize, discover, optimize, plot	Individual, social, and environmental simultaneously	SWPS and system-level outcome data

As shown in Table 1, paneugenesis does not replace existing models but integrates and extends them by specifying a unified, testable mechanism for generating reinforcing, cross-domain, better-than-before outcomes through intentional system design. Within this framework, regeneration is defined as the intentional creation of conditions that improve systems rather than merely restoring or sustaining them (20, 21). Preventive approaches reduce harm, whereas regenerative approaches produce measurable improvement.

While these frameworks provide important conceptual and practical contributions, paneugenesis differs in a critical way: it specifies a testable system design mechanism linking outcome definition, behavioral precursors, process optimization, and feedback measurement into a single integrated model. Most existing frameworks describe what influences health or what should be done, whereas paneugenesis specifies how systems can be deliberately engineered to generate reinforcing, cross-domain, better-than-before outcomes.

## 3 The evolution of health promotion toward regeneration

Health science and practice have advanced through a series of expanding models, each adding capabilities, thus building upon rather than replacing what came before. Early medical models focused primarily on diagnosing and treating disease. Prevention models extend this work by identifying and reducing risk factors before illness occurs. While prevention produced important gains, Wilde’s risk homeostasis theory illustrates a structural ceiling inherent to this approach: when perceived risk is reduced in one domain, compensatory behavior may restore prior risk levels elsewhere (28). This dynamic suggests that systems optimized primarily around harm reduction encounter self-limiting feedback that regenerative design directly addresses.

Health promotion broadened the scope further by encouraging protective behaviors and supportive environments (16). Salutogenesis marked a major conceptual advance by shifting attention from preventing disease to identifying and strengthening the origins and precursors of health (3–5). Rather than defining success as the absence of illness, salutogenesis defines health as a dynamic, resource-supported process shaped by coherence, meaning, and adaptive capacity. This shift moved the field toward health generation, rather than relying solely on problem avoidance.

Converging advances now support a further step: regeneration. Regenerative approaches do not merely aim to restore prior conditions or maintain current function; they aim to produce better-than-before outcomes by improving system capacity and reinforcing beneficial interactions over time (2, 20, 21). Regeneration, therefore, represents an expansion from promoting health within existing conditions to intentionally redesigning systems to produce healthier outcomes more reliably.

Paneugenesis is proposed as the operational framework for this next stage. It builds directly on treatment, prevention, promotion, and salutogenesis, and extends them through regenerative system design and measurable +3 outcomes. In this progression, earlier models remain valid and useful, but are incorporated into a broader generative framework that emphasizes continual improvement and net-positive impact.

## 4 Theoretical foundations of paneugenesis

Paneugenesis is constructed by integrating consistent advances across multiple established scientific traditions that explain how durable improvement is achieved in complex systems. Rather than replacing existing theory, it builds on and connects compatible findings from salutogenesis, quality management science, systems theory, complexity science, evolutionary biology, and behavioral science. Where these lines of work converge, they provide a coherent scientific basis for the design of regenerative health promotion.

### 4.1 Salutogenesis and health generation

Salutogenic theory marked a critical shift in health science by asking what generates health rather than what causes disease (3–5). Salutogenesis defines health as a dynamic condition supported by resources, coherence, and adaptive capacity, rather than as the absence of illness. Central to this model is the sense of coherence construct, comprehensibility, manageability, and meaningfulness, which predict resilience and well-being across populations.

Paneugenesis builds directly on this salutogenic foundation by shifting the focus from the origins of health to its generation through system design. While salutogenesis identifies health precursors, paneugenesis adds an operational design framework for intentionally strengthening those precursors through structured processes and feedback systems. In this way, paneugenesis serves as a regenerative extension of salutogenic logic.

### 4.2 Quality management and systems foundations

Quality management science shows that outcomes are determined by system structure and process design rather than by individual effort alone (2, 22). Variation, performance, and reliability are system properties. Sustainable improvement, therefore, requires continual system refinement guided by feedback, measurement, and iterative learning cycles.

Paneugenesis incorporates this principle by treating health and well-being outcomes as products of behavioral and environmental systems. Its four-function model, idealized outcome specification, precursor identification, process optimization, and progress plotting parallels quality improvement cycles and applies them to health promotion. This alignment makes regenerative health design measurable, testable, and improvable rather than aspirational.

Systems theory and idealized design show that meaningful improvement begins by specifying the desired outcome and then designing backward from that aim (1). System performance depends on relationships and interactions among components rather than on isolated elements. Attempts to optimize parts without regard for the whole often degrade overall outcomes. Paneugenesis adopts this systems orientation by starting with explicitly operationalized idealized outcomes and designing processes that generate reinforcing benefits across domains. This whole-system perspective supports +3 outcome logic and avoids siloed optimization that causes downstream harm. Earlier conceptual work has illustrated this integration through the “public health tree” model, which maps assessment, policy development, research, and assurance onto an interconnected system structure that supports health generation (14). Paneugenesis extends this structural foundation by specifying testable mechanisms through which such systems generate reinforcing, measurable +3 outcomes.

The Social Ecological Model further established that health behavior is shaped by nested influences across individual, interpersonal, organizational, community, and policy levels (29, 30). While this model effectively describes where influences occur across ecological levels, it does not specify how to intentionally design interactions across those levels to generate reinforcing rather than competing outcomes. Paneugenesis addresses this gap by operationalizing interbeneficence across all ecological levels simultaneously, specifying not only that multi-level influences exist, but how each level can be designed to strengthen the others. In this way, paneugenesis extends the Social Ecological Model from description toward coordinated regenerative system design.

### 4.3 Complexity, evolution, and interbeneficence

Complexity science shows that open systems develop higher levels of order when energy and information flows are properly organized (8). Order is not maintained passively; it is produced through structured exchanges and adaptive feedback. Living systems sustain themselves through continuous energy input and dynamic reorganization. Paneugenesis applies this behavioral and social principle: thriving requires energy-adding actions, supportive environments, and reinforcing feedback loops. Health-promoting behavior is therefore not merely protective but generative, contributing to upward developmental trajectories when system conditions support it.

Evolutionary and ecological science show that long-term progress often arises through cooperative and symbiotic relationships rather than competitive ones (13). Nonzero interaction theory further shows that mutually beneficial exchanges increase overall system capacity and stability over time (12). Paneugenesis incorporates this principle through the concept of interbeneficence, which denotes mutually reinforcing benefits among system participants. +3 outcomes operationalize interbeneficence by specifying that regenerative actions simultaneously strengthen personal, social, and environmental conditions rather than trading one for another.

Harari (10) extends this evolutionary logic into the human context, arguing that humanity has entered an era in which the defining challenge is no longer survival but the intentional design of systems that enable flourishing. Hertog (31), building on Hawking’s final theoretical work, further argues that the universe is participatory rather than predetermined, with properties and patterns emerging continuously through ongoing interaction and change. In this view, stagnation is structurally impossible; systems are continually co-created through action, making regeneration a practical design responsibility rather than an aspiration. In health promotion, the conditions for future well-being are actively shaped by present system design choices.

### 4.4 Behavioral and habit science

Behavioral science shows that durable behavior change depends less on intention alone and more on environmental structure, cues, reinforcement, and habit formation (6, 7). Social cognitive theory demonstrates that behavior emerges from reciprocal interactions among personal factors, environmental conditions, and behavioral patterns, and that self-efficacy beliefs are central to initiating and sustaining health-promoting action (32). Paneugenesis operationalizes this reciprocal logic by designing environments and feedback systems that strengthen efficacy through repeated regenerative experience rather than corrective instruction, making beneficial action progressively more automatic over time.

Prospect theory shows that losses loom larger than equivalent gains in human decision-making, meaning prevention-framed messaging, which implicitly emphasizes what could go wrong, carries a structural motivational disadvantage (33, 34). Crucially, this asymmetry does not simply mean that positive framing is preferable; it means that positive experiences must be substantially more frequent and powerful than negative ones to produce sustained behavioral change. Research on positive emotion and flourishing suggests that upward developmental spirals require positive experiences to substantially outweigh negative ones, with some estimates suggesting ratios of 3:1 or greater, before durable improvement takes hold (6). Paneugenesis directly addresses this threshold requirement by designing systems that generate reinforcing benefits across multiple domains simultaneously, so that the cumulative positive experience of regenerative action is strong enough and frequent enough to exceed the motivational weight of loss-oriented framing (59). Early empirical work in health promotion contexts similarly found that college students expressed significantly stronger intentions to engage in behaviors framed around health promotion than those framed around disease prevention, across all behavioral domains assessed, a preference that held regardless of the type of message they had received (35, 58).

### 4.5 Scientific convergence and regenerative design

Advances across multiple scientific domains converge on a consistent conclusion: durable improvement arises from strengthening system interactions and feedback structures rather than optimizing isolated components (1, 2). Systems science explains how interaction patterns produce outcomes. Salutogenesis clarifies which resources and precursors support health (3, 5). Evolutionary and symbiotic models show that long-term thriving depends on mutually reinforcing relationships (12, 13). Complexity science demonstrates that higher levels of order emerge in open systems when energy and information flows are properly organized (8). Behavioral science shows that habits and environments shape sustained action and upward developmental trajectories (6, 7). Together, these lines of evidence indicate that improvement is interaction-driven and design-dependent.

Recent analyses of human capability further argue that the defining challenge has shifted from scarcity management toward the intentional generation of benefit. Transformational progress in health systems has been linked to the redesign of institutions and professional systems to produce greater collective benefit (36). Bregman (37) similarly frames this shift as “moral ambition,” emphasizing the pursuit of substantial positive societal impact. Research on complex economic and governance systems shows that coordinated, polycentric approaches can generate reinforcing benefits across social systems when institutions are intentionally designed for shared outcomes (38). Bastani (39) and Klein and Thompson (40) similarly describe how emerging technological and economic systems may expand the potential for generating widespread benefit. This framing aligns directly with the central criterion of paneugenesis: that success in health promotion should be defined by measurable, better-than-before gains in engagement, well-being, and cross-domain capacity, rather than by the absence of problems.

Converging advances in regenerative design and systems science further show that achieving better-than-before outcomes is increasingly practical, not merely a distant ideal. Regenerative design frameworks demonstrate that human-built systems can be intentionally structured to produce net-positive effects, improving environmental and human conditions simultaneously, rather than simply reducing harm (20, 21). This represents a shift in design criteria from “less harmful” to “more beneficial,” paralleling the shift from prevention-centered to generative health models by defining success as measurable gains in capacity and well-being.

Energy systems research makes this design distinction explicit by contrasting hard and soft energy paths (18, 19). Hard paths, the traditional and prevailing methods, emphasize large, centralized, resource-intensive infrastructure optimized within existing paradigms. In contrast, soft paths prioritize efficiency, distributed resources, adaptability, and the reinforcement of system benefits. Analyses show that soft-path approaches can deliver greater total system benefit with fewer negative externalities when reinforcing efficiencies and distributed solutions are prioritized. This distinction illustrates a broader principle central to paneugenesis: outcomes are shaped by pathway design, not only by effort. Different system designs reliably produce different classes of outcomes.

Recent cross-field analyses further argue that advances in knowledge, technology, and coordination capacity expand what is achievable for human well-being when guided by generative design principles (39, 40). In this scientific context, regeneration is best understood as a practical design direction that creates conditions measurably better than before, rather than a distant possibility. Paneugenesis brings this regenerative design logic into health promotion by specifying behavior-linked, measurable system criteria that produce reinforcing gains in well-being, engagement, and cross-domain benefits.

Nature further confirms the logic of regenerative systems. Living systems maintain resilience and adaptability through feedback, cooperation, redundancy, and reciprocal exchange rather than linear control. Open-systems theory shows that adaptive order can emerge through self-organization when energy and information are appropriately structured (8). Biomimicry examines how these regenerative strategies can inform human design (17). Networked ecological systems demonstrate cooperative resource exchange and signaling that enhance collective resilience and long-term viability (41). Paneugenesis can therefore be understood as a biomimetic systems framework for health promotion, translating regenerative principles observed in living systems into intentionally designed human behavioral and social systems that generate reinforcing +3 outcomes.

## 5 Paneugenesis mechanism model: four functions for regenerative system design

**Figure 2:**
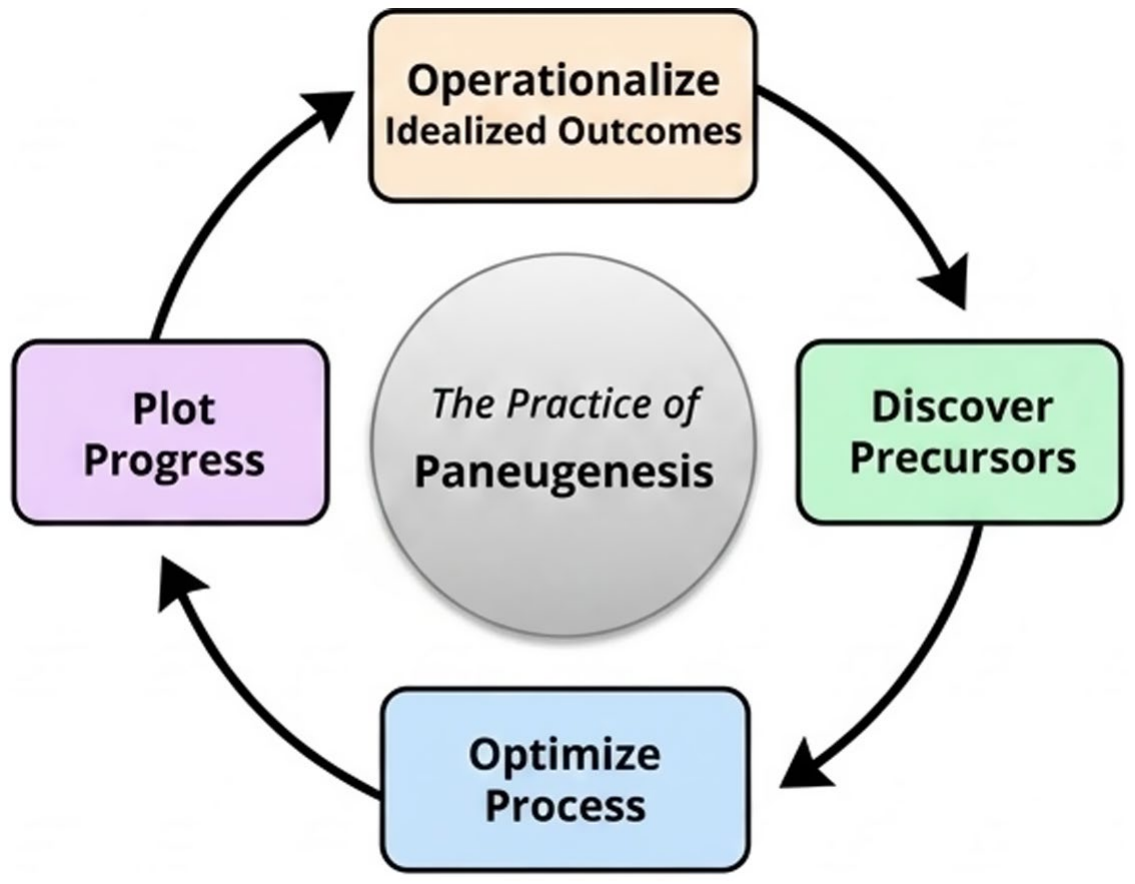
The practice of paneugenesis: a four-function regenerative cycle. Paneugenesis is operationalized through a four- function, iterative cycle that translates regenerative theory into actionable system design. These functions: operationalizing idealized outcomes, discovering precursors, optimizing processes, and plotting progress, form a continuous feedback system that produces and reinforces better-than-before outcomes across domains.

The central hypothesis of this article is that health promotion systems intentionally designed using the paneugenesis four-function model will generate greater long-term improvements in well-being, engagement, and cross-domain outcomes than systems designed primarily around risk reduction or problem control. Rather than functioning as a general philosophy, paneugenesis specifies a repeatable four-function mechanism model for designing, strengthening, and measuring regenerative systems (Figure 2).

This model builds directly on Deming’s quality management principle that process produce outcomes, and must be improved through structured feedback and continual refinement (2), and on Antonovsky’s salutogenic model that health emerges from strengthening precursors and resources rather than focusing only on disease avoidance (3, 5). Paneugenesis integrates and extends these foundations by specifying how ideal outcomes are operationalized, precursors are identified, processes are optimized, and progress is plotted in regenerative terms. The four-function model conceptually aligns with prior applications in public health contexts proposed to operationalize chronic wellness through assessment, policy development, research, and evaluation, and assurance functions (14).

The four functions are iterative and mutually reinforcing rather than strictly linear. Together, they form a regenerative design and improvement cycle.

### 5.1 Operationalize the idealized outcome

Paneugenesis begins by explicitly specifying the better-than-before outcome to be created. The idealized outcome is expressed in positive, generative terms and includes cross-domain benefit criteria rather than single-metric targets (1).

In paneugenesis, idealized outcomes are defined using +3 criteria, benefit for self, others, and the environment, ensuring that improvement targets are mutually reinforcing rather than tradeoff-based. This operationalization aligns with earlier calls for assessment processes that identify and define idealized health outcomes as a basis for system design (14).

For +3 outcomes, each domain requires distinct but complementary indicators. The individual domain (self) is assessed through validated measures of health-promoting behavior engagement, perceived health, life satisfaction, and sense of coherence, with the SWPS providing a validated operational instrument (23, 24). The social domain (others) is assessed through indicators of relational quality, social cohesion, civic engagement, and community capacity, consistent with social ecological measurement frameworks (29, 30). The environmental domain is assessed through measures of ecological impact, resource sustainability, and environmental quality relevant to the system context, consistent with regenerative design criteria (20, 21). To enable integrated evaluation, composite +3 indices can be constructed by combining standardized change scores across individual, social, and environmental domains, allowing for simultaneous assessment of domain-specific improvement and cross-domain reinforcement.

When trade-offs arise, where gains in one domain appear to come at cost to another, paneugenesis treats this as a signal that the system design requires further optimization rather than as an acceptable outcome. The goal of +3 criteria is not to mandate equal gains across all three domains in every cycle, but to ensure that no domain is systematically sacrificed and that reinforcing interactions across domains are actively sought through iterative process refinement.

For empirical testing, +3 outcomes should be operationalized as measurable change scores across domains over time. Paneugenesis predicts not only improvement within each domain, but positive covariance across domains, indicating reinforcing rather than substitution effects. For example:

- Individual: Change in SWPS scores, perceived health, or life satisfaction.
- Social: Change in network density, civic participation, or social cohesion.
- Environmental: Change in sustainability indicators, e.g., resource use or quality metrics.

Paneugenesis hypotheses can be tested using multiple study designs, including:

- Quasi-experimental designs comparing paneugenesis-based interventions to traditional interventions.
- Longitudinal cohort studies tracking engagement and outcome trajectories.
- Difference-in-differences models evaluating policy or systems-level implementation.
- Multi-level modeling to assess cross-domain effects.

These approaches allow for evaluation of both within-domain improvement and cross-domain reinforcement, which is a defining prediction of the model.

### 5.2 Discover the precursors

Once the idealized outcome is specified, paneugenesis identifies the behavioral, environmental, relational, and structural precursors that reliably produce that outcome. This step directly extends salutogenic logic by focusing on the origins and generators of health rather than on risk factors alone (3, 5).

Precursors include health-promoting behaviors, supportive environmental conditions, reinforcing social interactions, and meaning-supporting structures. This precursor orientation shifts from a traditional problem-reaction orientation to one of capacity generation. Paneugenesis asks what to strengthen and multiply, rather than only what to reduce. This precursor-focused approach is consistent with prior recommendations to identify the determinants of positive health outcomes rather than focusing exclusively on risk factors (14).

### 5.3 Optimize the process

Paneugenesis next applies quality improvement principles to strengthen the systems and processes that produce the identified precursors. Because outcomes are system-produced, durable improvement requires process redesign, feedback loops, and supportive structures rather than relying solely on individual motivation (2).

This distinction also clarifies the difference between creativity and innovation. As Gerber (42) observed, innovation involves doing new things, whereas creativity involves generating new ideas. Health promotion has generated many creative ideas, yet far fewer systems capable of reliably translating those ideas into sustained action. Paneugenesis explicitly addresses this gap by focusing on system design and execution, therefore ensuring that regenerative behaviors are repeatedly enacted through optimized processes, not merely imagined.

Process optimization involves adjusting environmental cues, supports, incentives, defaults, and feedback mechanisms to make regenerative behaviors easier to perform, repeat, and sustain. Behavioral science shows that habits form through structured repetition supported by context and reinforcement, not intention alone (7, 57). Positive emotion and meaningful engagement further broaden behavioral repertoires and support upward developmental spirals (6).

In paneugenesis, optimization is evaluated by reinforcing benefits, whether process changes increase the likelihood of +3 outcomes and mutually beneficial system interactions, rather than only by efficiency. This reflects a shift from managing conditions to designing systems that generate improved outcomes through structured interactions and supportive environments.

### 5.4 Plot progress

The fourth function is plotting progress through regenerative measurement and feedback. Paneugenesis emphasizes progress plotting rather than compliance tracking, consistent with quality improvement and learning system models (2). Measurement focuses on directional improvement in precursors, engagement, and reinforcing outcomes rather than solely on the absence of problems. In this sense, plotting progress within paneugenesis parallels improvements in decision-making that occur when intuitive judgment is supplemented or replaced by structured feedback and evidence-based models (33). The practical impact of such data-driven decision systems has been widely illustrated in domains ranging from sports analytics to behavioral decision science (43, 60). Progress plotting enables practitioners to treat each measurement cycle as a learning opportunity, refining both the system design and the understanding of which precursors are most generative in a given context, rather than evaluating programs against fixed endpoints.

This self-correcting capacity is itself a hallmark of adaptive systems: Harari (10) argues that humanity’s most significant advances have come from developing mechanisms that identify errors and improve iteratively, and not from executing perfect plans. Paneugenesis embeds this logic structurally; progress plotting is a continuous self-correction mechanism built into the design of regenerative systems; it is not an evaluation endpoint.

Validated instruments that assess health-promoting engagement and system-supporting behaviors, such as the SWPS, provide operational indicators aligned with this regenerative measurement approach (23–25, 44). Plotting progress makes improvement visible, supports adaptive adjustment, and reinforces continued participation.

### 5.5 Mechanism predictions and testability

Paneugenesis specifies outcome criteria, precursors, process variables, and measurement indicators, thereby generating formally testable hypotheses. The following hypotheses are testable within current methodological frameworks. Existing empirical findings are presented where relevant to support the plausibility of key constructs, measures, and relationships; however, these findings do not constitute direct tests of the full paneugenesis model. Importantly, supporting evidence presented here reflects validation of measurement constructs and related behavioral relationships, rather than direct empirical confirmation of the full paneugenesis system design mechanism. Where possible, supporting evidence is presented both for the measurement constructs used in evaluation and for behavioral or system-level mechanisms relevant to the proposed model.

*Hypothesis 1 (Engagement)*: Participants enrolled in health promotion programs explicitly designed to operationalize the four paneugenesis functions (idealized outcomes defined *a priori*, empirically identified behavioral precursors, structured process optimization, and longitudinal progress tracking) will demonstrate significantly greater increases in SWPS scores from baseline to 6- and 12-month follow-up compared to participants enrolled in programs primarily structured around risk reduction or harm avoidance.

*Supporting evidence for model components*: Prior studies (*n* = 2,140; n = 691, respectively) demonstrate that higher engagement in health-promoting behaviors, as assessed by the SWPS, is consistently associated with better perceived health and academic performance (24, 45). These findings support the validity of the engagement constructs and measurement approach used to evaluate paneugenesis. However, they do not constitute a direct test of the four-function paneugenesis model. Rather, they demonstrate that the behavioral engagement constructs targeted by the model are empirically associated with improved health and performance outcomes, providing a plausible empirical basis for evaluating the proposed system design mechanism.

*Hypothesis 2 (Well-being)*: Individuals who engage in paneugenesis-identified precursor behaviors will demonstrate significantly greater increases from baseline to follow-up in life satisfaction, perceived health status, and sense of coherence compared to individuals participating in single-domain behavior change interventions, controlling for baseline levels of each outcome.

*Supporting evidence for model components*: Participants grouped by health-promoting behavior engagement showed linear, statistically significant differences in perceived health across high (*M* = 8.64), middle (*M* = 7.98), and low (*M* = 6.22) groups (*p* < 0.001), with all three groups distinguishable, a sensitivity advantage that illness-based measures alone could not achieve, as those discriminated only between the highest and lowest groups (46). These findings support the plausibility of precursor-focused improvement, a central mechanism in paneugenesis. However, they do not constitute a direct test of the full four-function system design. Instead, they provide empirical evidence that differences in engagement with health-promoting behaviors are associated with measurable differences in well-being outcomes, supporting the premise that identifying and strengthening behavioral precursors can generate improved health outcomes.

*Hypothesis 3 (Cross-domain benefit)*: If systems are designed using +3 outcome criteria, simultaneously targeting individual, social, and environmental benefit, then evaluation data will demonstrate reinforcing rather than substitutive improvement across domains, such that gains in one domain predict rather than trade off against gains in others. These domains can be operationalized through independent indicators of individual well-being (e.g., SWPS scores, perceived health, life satisfaction), social contribution and relational outcomes (e.g., collaboration, community engagement), and environmental stewardship behaviors or sustainability indicators.

*Supporting evidence for model components*: Prior SWPS research demonstrates that domain scores exhibit distinct but correlated predictive patterns: physical and emotional dimensions predict perceived health, while the vocational dimension predicts academic performance. In addition, perceived health and academic performance are significantly correlated (r = 0.111, *p* < 0.004), indicating that engagement across multiple domains is associated with reinforcing rather than competing outcomes (24). These findings support the plausibility of cross-domain reinforcement, a central feature of the +3 design logic. However, they do not constitute a direct test of the paneugenesis model itself, which predicts that intentional system design will produce reinforcing improvements across individual, social, and environmental domains.

*Hypothesis 4 (Maintenance)*: Participants in interventions that embed target behaviors through environmental redesign and structured feedback loops will demonstrate significantly greater maintenance of behavioral outcomes between post-intervention and 12-month follow-up compared to participants in interventions relying primarily on awareness-building, education, or intention-based strategies.

*Supporting evidence for model components*: The Salutogenic Wellness Promotion Scale (SWPS) demonstrates moderate to substantial test–retest reliability (average ICC = 0.55; range 0.47–0.75 across dimensions) over a 14-day interval (47), indicating that the scale is sufficiently stable to detect meaningful behavioral change over time. These findings support the suitability of the measurement framework for evaluating behavioral change and maintenance. However, they do not constitute a direct test of the paneugenesis model’s process optimization mechanism. Rather, they establish that the measurement framework is capable of detecting maintenance effects when regenerative behaviors are embedded through environmental redesign and feedback processes.

*Hypothesis 5 (Diffusion)*: In high-visibility implementation settings where early adopters produce observable +3 outcomes, the proportion of new adopters within the associated social network over a defined observation period will be significantly greater than in comparable settings without visible early adopters, consistent with diffusion-of-innovation mechanisms. Supporting evidence for model components: Diffusion of innovations theory and tipping point research demonstrate that visible early adopters and socially connected individuals accelerate the adoption of behavior when benefits are observable and credible (48–51).

These hypotheses can be tested using longitudinal cohort designs, quasi-experimental comparisons, or randomized controlled trials where feasible across varied health promotion contexts. Analytic approaches may include multilevel modeling to assess cross-domain effects, structural equation modeling to examine relationships among precursors and outcomes, and difference-in-differences designs to compare paneugenesis-based interventions with traditional risk-reduction programs. Because the four-function model specifies both mechanism and measurement approach, it is structured for confirmatory testing and iterative refinement. Disconfirmation of any individual hypothesis would provide actionable guidance for model refinement, consistent with quality improvement principles emphasizing iterative learning and system improvement (2).

Paneugenesis aligns with existing measurement tools (e.g., SWPS, well-being scales, system-level indicators), therefore, it can be tested without requiring entirely new measurement infrastructure, increasing its practical feasibility for research and applied settings. The evidence presented supports key components of the model, including behavioral precursors, engagement measures, and system feedback processes. However, direct empirical validation of the full paneugenesis model as an integrated system has not yet been conducted, and remains a central priority for future research.

## 6 Implications for health promotion research and practice

Paneugenesis advances health promotion by reframing success criteria as better-than-before gains in system capacity, engagement, and cross-domain benefits rather than defining success solely in terms of risk reduction. By integrating salutogenic principles, quality management logic, systems science, and regenerative design, paneugenesis provides a structured, integrated framework for generating durable improvements rather than isolated intervention effects.

### 6.1 Implications for research

Paneugenesis introduces several research directions that extend current health promotion inquiry.

First, it shifts the emphasis of primary outcomes toward generative metrics rather than reduction-focused metrics. While prevention remains valuable, paneugenesis predicts that systems evaluated on strengthening precursors and reinforcing +3 outcomes will demonstrate stronger sustained gains in well-being and engagement than systems evaluated primarily on harm reduction. An additional research question concerns whether regenerative system design produces prevention as a secondary effect. If paneugenesis is correct, systems designed to strengthen health-promoting engagement, well-being, and system capacity should also produce reductions in risk factors and health problems over time, even when prevention is not the primary design objective. This possibility reflects a key theoretical prediction of the model: generating health capacity may yield prevention as an emergent by-product. Comparative studies that evaluate both well-being gains and risk reduction outcomes across intervention designs would therefore provide an important test of regenerative health promotion. If supported empirically, this finding would suggest that health generation may represent a more efficient pathway to prevention than prevention-focused strategies alone.

Second, the four-function mechanism model provides a testable framework for intervention design. Research can examine whether explicitly operationalizing idealized outcomes, identifying precursors, optimizing processes, and plotting progress produce greater behavioral adherence and outcome stability than traditional program models.

Third, paneugenesis supports multi-level evaluation. Because the model incorporates individual behaviors, environmental supports, relational dynamics, and structural design, it encourages measurement across ecological levels rather than limiting assessment to individual-level change.

Fourth, regenerative measurement approaches, such as tracking improvements in health-promoting engagement, can be used to examine whether generative indicators predict long-term resilience and adaptive capacity beyond short-term risk reduction metrics (23–25).

Fifth, paneugenesis shifts the focus from addressing problems to generating reinforcing strengths across populations. This shift has important implications for health inequalities. The Inequality Paradox (52) shows that population-level interventions can improve overall health while widening disparities when benefits are disproportionately realized by already advantaged groups. Paneugenesis predicts that interventions designed to generate +3 outcomes will produce greater gains among individuals and groups with lower baseline levels of health-promoting engagement, such that improvements in outcomes (e.g., SWPS scores, well-being indicators) are larger in higher-need populations. This pattern of differential improvement is expected to reduce disparities over time, rather than maintain or widen them. This prediction reflects the expectation that strengthening foundational capacities produces the greatest improvements among those starting with the lowest levels of health-promoting engagement. Future research should test whether paneugenesis-based interventions produce greater relative gains among lower-resourced or lower-baseline groups compared to traditional intervention models.

Collectively, these directions move health promotion research toward examining how systems produce upward developmental trajectories rather than focusing solely on downstream outcomes.

### 6.2 Implications for practice

In practice settings, paneugenesis offers a structured approach to designing interventions, organizations, and policies that yield reinforcing benefits rather than isolated gains.

Programs guided by paneugenesis would define success using better-than-before criteria, identify and strengthen salutogenic precursors, redesign environments to support reinforcing habits, use feedback systems to make progress visible, and evaluate impact across personal, social, and environmental domains.

This approach does not eliminate the need for prevention or treatment strategies. Instead, it situates them within a broader regenerative framework in which prevention is often a byproduct of strengthened health capacity rather than the primary driver of improvement.

For practitioners, this reframing shifts emphasis toward multiplying assets and expanding capability rather than controlling risk or correcting deficits. Because paneugenesis incorporates continual improvement logic, it is compatible with existing quality improvement structures already used in healthcare and community health systems.

### 6.3 Implications for policy and system design

Systems thinking approaches have been increasingly applied to health policy and system design to support coordinated, multi-level improvement (53). Policy environments often operate under conditions of constrained authority, fragmented governance, and competing priorities (54). Paneugenesis is explicitly compatible with these realities by emphasizing policy layering rather than replacement, embedding regenerative criteria within existing regulatory and planning frameworks. For example, jurisdictions unable to implement comprehensive food system reform may integrate food access considerations into transportation or climate adaptation policies, producing meaningful system improvements. This approach demonstrates that regenerative system change can be created through incremental, reinforcing alignment across sectors over time, even within existing constraints.

Regenerative system change does not require universal adoption to achieve broad impact. As proposed in Hypothesis 5, regenerative systems that generate visible +3 outcomes may diffuse through social networks as early actors demonstrate credible advantages. Research on social diffusion demonstrates that behaviors and innovations spread most effectively once early actors establish credibility, observability, and demonstrated relative advantage (50). Tipping point dynamics further show that transformation accelerates when a small number of highly connected or motivated individuals act in visible, coordinated ways that others find compelling and replicable (48, 49). Paneugenesis aligns with this logic: regenerative system design does not depend on simultaneous population-wide adoption. It requires intentional early actors who generate visible +3 outcomes, making the benefits of regenerative behavior observable, credible, and attractive. Policy design can leverage this by identifying and supporting high-visibility contexts where regenerative systems can clearly demonstrate their advantages.

This framework aligns with ecological and interbeneficence principles by recognizing that long-term health depends on mutually reinforcing relationships among systems. By explicitly specifying +3 outcome criteria, paneugenesis provides policymakers with a design lens that encourages alignment across sectors rather than siloed optimization. This integration of policy layering, systems design, and diffusion dynamics positions regenerative approaches within established implementation science and systems thinking frameworks, while extending them through a structured focus on multi-domain benefit generation.

### 6.4 Contribution to health promotion theory

The theoretical contribution of paneugenesis lies in specifying a systems design mechanism through which reinforcing improvements in health, social, and environmental outcomes can be intentionally generated. Paneugenesis contributes to health promotion theory by integrating insights from salutogenesis, quality management systems, behavioral science, and complexity science into a unified framework for intentionally generating reinforcing improvements in health, social, and environmental outcomes.

By defining regeneration as better-than-before system performance and specifying operational design functions, paneugenesis offers a theory that is grounded in established science, structured for empirical testing, compatible with ecological and systems models, adaptable across contexts, and oriented toward sustained, reinforcing improvement.

In doing so, paneugenesis proposes a next-stage development in health promotion theory that emphasizes intentional design of systems capable of generating expanding benefit over time.

## 7 Discussion: strengths, limitations, and future directions

Paneugenesis is proposed as a regenerative extension of established health promotion science, integrating salutogenesis, quality systems theory, complexity science, behavioral science, and regenerative design into a structured four-function model. Its primary strength lies in its integrative coherence. The contribution of paneugenesis lies not only in its conceptual integration, but in its specification of measurable outcomes, testable mechanisms, and adaptable implementation pathways across both ideal and constrained system conditions. Paneugenesis synthesizes compatible advances across disciplines into an operational framework for producing better-than-before outcomes, rather than by introducing an entirely new theoretical base.

The conceptual foundations of paneugenesis are consistent with prior models that emphasized chronic wellness, health-promoting ecosystems, and systems-level integration of public health functions, including salutogenic theory, social ecological models of health promotion, and integrated systems approaches to population health (4, 14, 30, 55). These earlier frameworks provided important structural and conceptual advances but did not specify a testable mechanism for generating reinforcing, cross-domain outcomes. Paneugenesis builds on this foundation by integrating these concepts into a unified, empirically testable model grounded in established theories from salutogenesis, quality management, behavioral science, and complexity science.

While selected conceptual and popular texts are used to illustrate broader framing and emerging directions, the proposed mechanism model is grounded in established, peer-reviewed theoretical foundations, including salutogenesis, quality management systems theory, behavioral science, and complexity science.

### 7.1 Strengths of the framework

One strength of paneugenesis is its explicit mechanism structure. By specifying idealized outcome operationalization, precursor identification, process optimization, and progress plotting, the framework moves beyond conceptual aspiration toward testable design criteria. This structure allows researchers and practitioners to examine not only whether outcomes change, but how system features produce those changes.

A second strength is its alignment with validated measurement tools. The emphasis on precursor strengthening and engagement-based metrics allows for empirical testing using existing salutogenic instruments, including measures of health-promoting behavior and well-being (23–25). This strengthens the feasibility of operationalization.

A third strength is cross-domain applicability. Because paneugenesis incorporates +3 criteria, benefit for self, others, and the environment, it is adaptable across individual, organizational, and policy contexts while maintaining consistent design logic.

### 7.2 Limitations and scope

Paneugenesis is currently a theory and structured framework rather than a fully validated intervention model. While elements of its precursor measurement logic have empirical support, comprehensive longitudinal testing of the full four-function model remains an area for future research.

Paneugenesis operates effectively within real-world system conditions, where funding limitations, regulatory constraints, and governance structures shape what is possible. Within these realities, it emphasizes strategic adaptation while enabling transformation over time, embedding regenerative (+3) criteria within existing structures. Regeneration includes both incremental and transformative change, consistent with Deming’s principle that continual improvement drives system transformation. In practice, this may occur through iterative modification of existing processes, including leveraging mandated planning structures, embedding +3 criteria into existing programs, and using community-generated data to influence institutional priorities. Even without full system control, targeted improvements in precursor conditions (e.g., access, social reinforcement, or behavioral cues) can generate localized +3 outcomes that accumulate and expand. These reinforcing gains can contribute to broader system transformation over time and align with diffusion mechanisms, where visible early improvements increase adoption despite structural constraints.

Paneugenesis should also be interpreted in relation to existing theoretical frameworks that promote well-being and behavioral change, including positive psychology and Self-Determination Theory (SDT). Interventions grounded in SDT, which emphasize autonomy, competence, and relatedness, have demonstrated effectiveness in supporting sustained motivation and behavioral engagement (56). The paneugenesis model does not replace these approaches but instead integrates such behavioral mechanisms within a broader systems design framework. Specifically, paneugenesis focuses on intentionally structuring environments, feedback processes, and cross-domain outcome criteria so that health-promoting behaviors generate reinforcing benefits simultaneously for individuals, others, and environmental systems. Whether regenerative system design produces greater or more durable outcomes than well-implemented SDT-based or other positive psychology interventions remains an empirical question that future studies should directly test.

Paneugenesis operates within real-world system conditions, where structural constraints, policy environments, and competing priorities shape what can be implemented at any given time. Within these contexts, regenerative change can be initiated through partial system redesign, including alignment of incentives, environments, and behaviors at multiple levels. Future research should examine how contextual factors influence the adoption, scaling, and sustainability of regenerative system design.

Although the +3 framework is designed to produce reinforcing, cross-domain benefits, complex systems require intentional alignment to ensure that gains remain mutually reinforcing rather than fragmented. Further work is needed to clarify how these reinforcing relationships can be consistently achieved and sustained across diverse contexts.

Paneugenesis complements, rather than replaces, existing approaches to treatment and prevention. Acute care and risk reduction remain essential components of health systems; paneugenesis extends these efforts by providing a generative framework for creating better-than-before outcomes through continual improvement.

### 7.3 Future research directions

Future empirical research should evaluate several core predictions of paneugenesis. These include whether operationalizing idealized outcomes improves durability. Whether precursor-focused measurement better predicts long-term resilience. And whether the four-function model supports sustained behavioral gains.

Additional research should examine whether paneugenesis-based interventions produce greater improvements among lower-baseline populations. Reductions in health inequalities are expected to emerge as a by-product of strengthening foundational capacities, rather than as the primary intervention target. Research should also assess whether +3 outcome criteria improve cross-sector policy alignment and multi-level health outcomes.

Longitudinal and comparative study designs will be important for testing these predictions and assessing how system components emerge, interact, and strengthen over time. Implementation science approaches can evaluate how paneugenesis performs across diverse contexts and populations.

Research on social diffusion should examine how regenerative practices spread. This includes whether early, visible implementation triggers broader adoption, consistent with diffusion theory (48–50).

Over time, iterative empirical refinement can strengthen, adapt, and extend the model. In keeping with quality improvement principles, paneugenesis should be understood as a continually improving framework supported by structured evaluation and feedback.

## 8 Conclusion

Health promotion science has progressively expanded from treatment to prevention to promotion and salutogenesis. Converging advances across systems science, quality management, evolutionary theory, behavioral science, and regenerative design now support a further step: intentional design of systems capable of producing better-than-before outcomes. Transformational progress in health systems often requires aiming to generate substantial societal benefit rather than simply managing existing problems (36). Bregman (37) similarly describes this orientation as “moral ambition,” emphasizing the pursuit of extraordinary good. Paneugenesis is proposed as a regenerative extension of this progression in health promotion science. Popular texts are used for framing; the mechanism rests on a grounded approach in systems science, salutogenesis, quality management, and behavioral/complexity theory.

By integrating salutogenic principles with quality system logic and interbeneficence-based design criteria, paneugenesis provides a structured four-function mechanism for operationalizing idealized outcomes, strengthening precursors, optimizing processes, and plotting progress through measurable feedback. This framework reframes success as reinforcing gains in engagement, well-being, and cross-domain benefits rather than as risk reduction alone. Because paneugenesis specifies operational design functions and measurable indicators, it is structured for empirical testing and iterative refinement. Its contribution lies not in replacing existing health promotion models, but in synthesizing compatible advances into a coherent regenerative framework capable of producing durable, reinforcing improvement.

As scientific understanding of complex systems continues to advance, health promotion can move toward intentionally generating expanding benefit, thus moving beyond maintaining equilibrium. Paneugenesis offers one structured pathway for translating convergent science into regenerative practice, supporting healthier individuals, stronger communities, and more resilient environments through measurable, continual improvement.

## Data Availability

The original contributions presented in the study are included in the article/supplementary material, further inquiries can be directed to the corresponding author.

## Footnotes

1 This framework is also informed by lived experience. Following a near-fatal head-on collision in adolescence, recovery did not occur through restoring prior conditions, which was not possible, but through the gradual development of small, repeatable actions that expanded what was possible over time. The result was not a return to a previous state, but the creation of a different and ultimately improved trajectory. This experience illustrates a core premise of regeneration: progress is not achieved by undoing the past, but by intentionally building new capacity. While this account is illustrative rather than evidentiary, it is included to clarify the conceptual mechanism in a manner consistent with systems and behavior-change theory.
